# The complete chloroplast genome of *Pholidota imbricata* (Orchidaceae)

**DOI:** 10.1080/23802359.2019.1675549

**Published:** 2019-10-11

**Authors:** Wen-Hui Rao, Jie Huang, Li-Qiang Li, Ting-Zhang Li, De-Min Kong, Jian-Bing Chen

**Affiliations:** a Key Laboratory of National Forestry and Grassland Administration for Orchid Conservation and Utilization, Shenzhen, Guangdong, China;; b Shenzhen Key Laboratory for Orchid Conservation and Utilization, The National Orchid Conservation Centre of China, and The Orchid Conservation and Research Centre of Shenzhen, Shenzhen, Guangdong, China

**Keywords:** *Pholidota imbricata*;chloroplast genome;phylogenetic, Coelogninae, Orchidaceae

## Abstract

*Pholidota imbricata* belongs to tribe Coelogninae in Orchidaceae distributed in Sichuan, Xizang, and Yunnan. Here, we report the first complete chloroplast (cp) genome and the cp genome features of *P. imbricata.* The complete cp genome sequence of *P. imbricata* is 159,292 bp in length and presented a typical quadripartite structure including one large single-copy region (LSC, 87,515 bp), one small single-copy region (SSC, 20,999 bp), and two inverted repeat regions (IRs, 25,389 bp each). The cp genome encoded 141 genes, of which 108 were unique genes (80 protein-coding genes, 24 tRNAs, and 4 rRNAs). The phylogenetic relationships show that *P. imbricata* is sister to the species of the genus *Pleione* in tribe Coelogninae.

The genus *Pholidota* belongs to the tribe Coelogninae (Orchidaceae: Epidendroideae) with 12 species, ranging through mainland and SE Asia, Australia, New Guinea, and the Pacific islands. (Chen et al. 2009; Pridgeon et al. 2014). Plants of *Pholidota* are widely used in Chinese traditional medicine (Zou et al. 2017; Fu et al. 2018; Huang et al. 2018; Zhang et al. 2018). The complete chloroplast (cp) genome sequence of *P. imbricata* was assembled in this study.

Specimens of *P. imbricata* were deposited in the National Orchid Conservation Centre herbarium (NOCC; specimen code Z.J.Liu 3524; geospatial coordinates N22°36′1″ E114°10′42″). Total genomic DNA was extracted from fresh material using the modified CTAB procedure of Doyle and Doyle (1987). Sequenced on Illumina Hiseq 2500 platform (Illumina, San Diego, CA). Genome sequences were screened out and assembled with MITObim v1.8 (Hahn et al. 2013), which resulted in a complete circular sequence of 159,292 bp in length. The cp-genome was annotated with CpGAVAS (Liu et al. 2012).

The cp genome sequence of *P. imbricata* (GenBank accession MN398392) is 159,292 bp long and presented a typical quadripartite structure including one large single-copy region (LSC, 87,515 bp), one small single-copy region (SSC, 20,999 bp), and two inverted repeat regions (IRs, 25,389 bp each). The cp genome encoded 141 genes, of which 108 were unique genes (80 protein-coding genes, 24 tRNAs, and 4 rRNAs).

Three accessions of Coelogninae species and two outgroups were used for the molecular analysis. The phylogenetic tree was constructed based on the maximum-likelihood (ML) methods. The ML analysis was performed using the CIPRES Science Gateway web server (RAxML-HPC2 on XSEDE 8.2.10) with 1000 bootstrap replicates and settings as described by Stamatakis et al. (2008). The result showed that *P. imbricata* is mostly distinguished from *Pleione* species (Figure 1). This newly reported cp genome will be helpful for further phylogenetic study, species identification, and genetic engineering.

**Figure 1.**
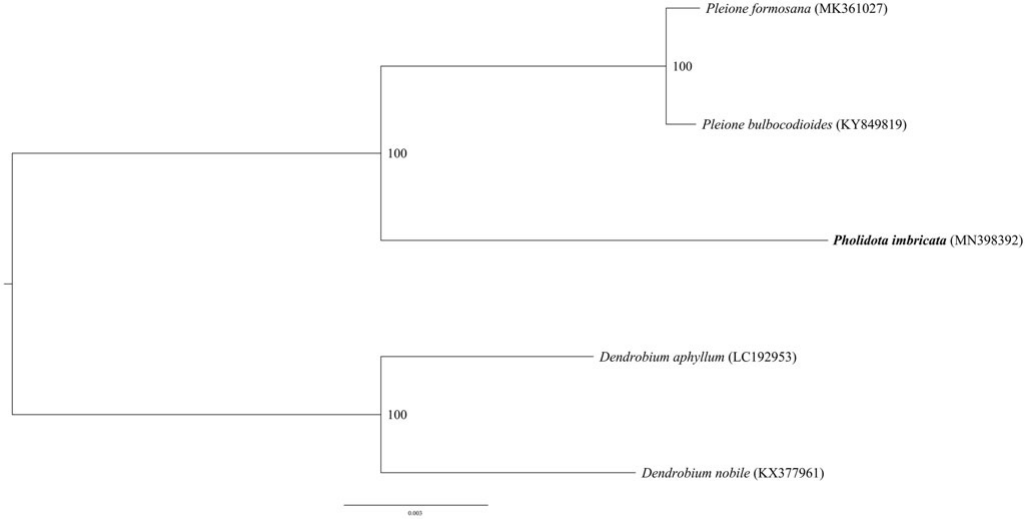
Phylogenetic position of *Pholidota imbricata* inferred by maximum-likelihood (ML) of complete cp genome. The bootstrap values are shown next to the nodes.

## References

[CIT0001] Chen SC, Liu ZJ, Zhu GH, Lang KY, Ji ZH, Luo YB, Jin XH, Cribb PJ, Wood JJ, Gale SW, et al. 2009 Orchidaceae In: Wu ZY., Raven PH, Hong D, editors. Flora of China, vol. 25. Beijing (China): Science Press; St. Louis (MO): Missouri Botanical Garden Press; p. 211–235.

[CIT0002] Doyle JJ, Doyle JL 1987 A rapid DNA isolation procedure from small quantities of fresh leaf tissue. Phytochem Bull. 19:11–15.

[CIT0003] Fu CD, Li XN, Yang WB, Zeng CQ, Guan SY 2018 Study on quality standard of *Pholidota imbricata* Lindl. J Guangdong Pharma Univ. 34(2):165–168.

[CIT0004] Hahn C, Bachmann L, Chevreux B 2013 Reconstructing mitochondrial genomes directly from genomic next-generation sequencing reads—a baiting and iterative mapping approach. Nucleic Acids Res. 41(13):e129. 23661685 10.1093/nar/gkt371 PMC3711436

[CIT0005] Huang MQ, Zhu YH, Zhao SY 2018 Effect of polysaccharide of *Pholidota imbricata* Lindl. on the expression of Th1/Th2 cytokines in mice infected with *Mycoplasma pneumoniae*. China Med Herald. 15–19.

[CIT0006] Liu C, Shi LC, Zhu YJ, Chen HM, Zhang JH, Lin XH, Guan XJ 2012 CpGAVAS, an integrated web server for the annotation, visualization, analysis, and GenBank submission of completely sequenced chloroplast genome sequences. BMC Genomics. 13(1):715. 23256920 10.1186/1471-2164-13-715 PMC3543216

[CIT0007] Pridgeon AM, Cribb PJ, Chase MW, Rasmussen FN 2014 Genera Orchidacearum. New York (NY): Oxford University Press; p. 1–544.

[CIT0008] Stamatakis A, Hoover P, Rougemont J 2008 A rapid bootstrap algorithm for the RAxML web-servers. Syst Biol. 75:758–771. 10.1080/10635150802429642 18853362

[CIT0009] Zhang M, Zhu H, Chen L, Li L, Dai ZH 2018 Study on pharmacognosy identification of *Pholidota imbricata* Lindl. Lishizhen Med Mate Ria Medica Res. 29(9):2177–2179.

[CIT0010] Zou ZL, Huang P, Zeng HT, Xu QX, Li JX, Liu YQ 2017 The research progress of Chinese *Pholidota* Pseudobulb or Herb’s. Biol Activ Funct. 44(2):61–62.

